# The hidden athlete: exploring work and performance ability of professional dancers through a mixed-method-approach

**DOI:** 10.1186/s13102-025-01367-0

**Published:** 2025-11-04

**Authors:** Hannah Sophia Hofmann, Cleo Kramer, Nina Marie Schmidt, Johanna Lachmann, Leon Trillmich, Matthias Sand, Ingo Froböse, Bianca Biallas

**Affiliations:** 1 Institute of Movement Therapy and Movement-Oriented Prevention and Rehabilitation, German Sport University Cologne, Cologne, Germany; 2 Department of Sport Science, University of Konstanz, Constance, Germany; 3 Survey Design and Methodology, GESIS – Leibniz-Institute for the Social Science, Mannheim, Germany

**Keywords:** Occupational Health, Athletic Performance, Work Environment, Elite Sport

## Abstract

**Background:**

Despite the high physical, psychological, and artistic demands, professional dancers still lack holistic and interdisciplinary workplace support comparable to elite sport. This study examines challenges and opportunities within the work ability framework for implementing measures in theaters to maintain work ability and enhance performance ability.

**Methods:**

An exploratory sequential mixed methods design (QUAL ➜ quan) was used. 25 guided interviews with n=14 leaders (47.1 ± 9.9 years) and n=11 professional dancers (30.2 ± 3.9 years) were conducted and analyzed using qualitative content analysis following Mayring. An online questionnaire survey of n=75 professional dancers (28.76 ± 7.74 years) with 62.7% female, 36% male, and 1.3% non-binary, was administered. The questionnaire consisted of sociodemographic data, the Work Ability Index (WAI) and items based on the results of the qualitative data, with respondents required to rank them. Factors influencing the work ability were tested using several generalized linear models and model comparisons as well as Kruskal–Wallis rank sum and t-test.

**Results:**

The primary challenges can be categorized as structural and personal (leaders/dancers). Emerging challenges address e.g., the definition of work and performance ability, work organization, preservation of tradition and interpersonal communication. WAI ranges from moderate (36%) to good (33.3%). Working as a Demi-soloist (Kruskal–Wallis chi-squared = 5.2141, df = 1, *p*-value = 0.0224), in a larger ensemble (Kruskal–Wallis chi-squared = 5.3075, df = 1, *p*-value = 0.02123) and/or within hierarchical structures (t = -1.7777, df = 52.038, *p*-value = 0.0813) has a negative impact on WAI. In the context of areas for improvement and development, the most prioritized item was ‘Support and care of the dancers comparable to elite sports‘ (49.3%).

**Conclusions:**

In order to integrate the importance of performance ability into existing company structures and adapt them accordingly, measures must be taken at both a structural and individual level. Contextual factors such as ensemble size and hierarchical structures need to be considered, with particular attention to vulnerable groups like demi-soloists when developing empowerment strategies. The model of integrated performance ability in professional dance reveals that performance ability is co-produced through continuous alignment between individual performers and institutional structures, necessitating interventions that address both personal capacity-building and organizational adaptation.

## Background

The everyday life of professional dancers has similarities with elite sport [1] as they must attain a high level of technical skill while also demonstrating expressiveness and artistic sensitivity [2]. Professional dancers are exposed to notably elevated physical stress, as studies indicate that the typical minimum of weekly training is 19.1 to 27.5 h per week [3]. The frequency of performances and events can lead to a significant increase in training time, as some individuals train up to 31.5 to 35.5 h per week. Studies have shown that elevated training volume is associated with an increased risk of injury, with 0.62–5.6 injuries occurring per 1.000 h of training [4, 5]. Such injuries significantly affect professional dancers’ performance and jeopardize their careers, which is also reflected in psychological issues [6, 7]. Even if professional dancers avoid injuries, the extended training load can lead to physical and mental fatigue [8–10]. This is due to the fact that many theaters often lack sufficient regeneration time and/or measures, limiting professional dancers’ ability to recover [3]. Along with the physical strain that professional dancers must endure, there is a considerable psychological burden for many artists, who have reported a lack of planning and job insecurity [11, 12]. To summarize, professional dancers engage in elite sport with an artistic intention in an occupational setting [13].

The objective in the field of elite sport is to provide the best possible environment to train and preparation to compete in local and global events. Specialized coaches, physiotherapists, nutritionists or sport psychologists provide support throughout the athlete's journey. The purpose is to enhance athletic performance and competitive success [14, 15]. In sport science, performance ability is defined as the complex interaction of multiple factors, including physiological, coordinative, psychological, tactical-cognitive, social, and genetic elements [16]. In contrast, the term work ability in the field of occupational work is defined as the alignment between an individual's resources and the demands of their occupation. This conceptualization considers the individual's distinctive potential and capabilities [17], as well as the broader social context [18]. Tempel and Ilmarinen [19] have developed a model named 'the house of work ability', comprising four distinct levels. Those are based on the first floor including performance ability (functional capacities) and health. The second floor represents competence, work experiences and learning, the third floor encompasses values, motivation, and attitude, and the fourth and final floor comprises work, work environment, work community and management. This floor is the largest and is supported by all the floors below [19, 20]. The work ability as the roof of the house declines with deteriorating working conditions, while improved work ability is associated with better working conditions and health. Reduced work ability can be found when there was a slight or significant increase in physical activity [21].

An examination of elite sport within the occupational setting reveals a discrepancy in the support provided to elite athletes and professional dancers which can be attributed to the divergent systems, activity settings, and objectives [22, 23]. So-called “dancer wellness programs” have been implemented, assessed, and their findings disseminated at numerous theaters in recent years. However, these programs are predominantly designed for the individual professional dancer [24]. This encompasses measures such as preventive screenings (including the development of monitoring apps), healthcare in the medical sense, knowledge transfer on health-related topics, and an additional offer of individual athletic training [25–27]. But a comprehensive health promotion program should consider intrapersonal aspects e.g., safety or health and place greater emphasis on institutional, systemic and interpersonal factors to promote social well-being and equity within a professional dancer wellness framework [24].

This is particularly relevant in the German theater system, which is characterized by a hierarchical organizational structure, a repertoire system, and a pronounced reliance on public funding. The artistic director occupies the highest leadership position within the organization and bears responsibility for major decisions. These subsequently shape the training regimen and practice intensity. This hierarchical system inherently centralizes decision-making authority, contributing to a structural imbalance of power that restricts participatory opportunities for other members [22]. To further explore the circumstances of professional dancers working in theaters, the objective of this study is to identify challenges and opportunities within the work ability framework for implementing measures in theaters to maintain work ability and enhance performance ability.

## Methods

**Fig. 1:**
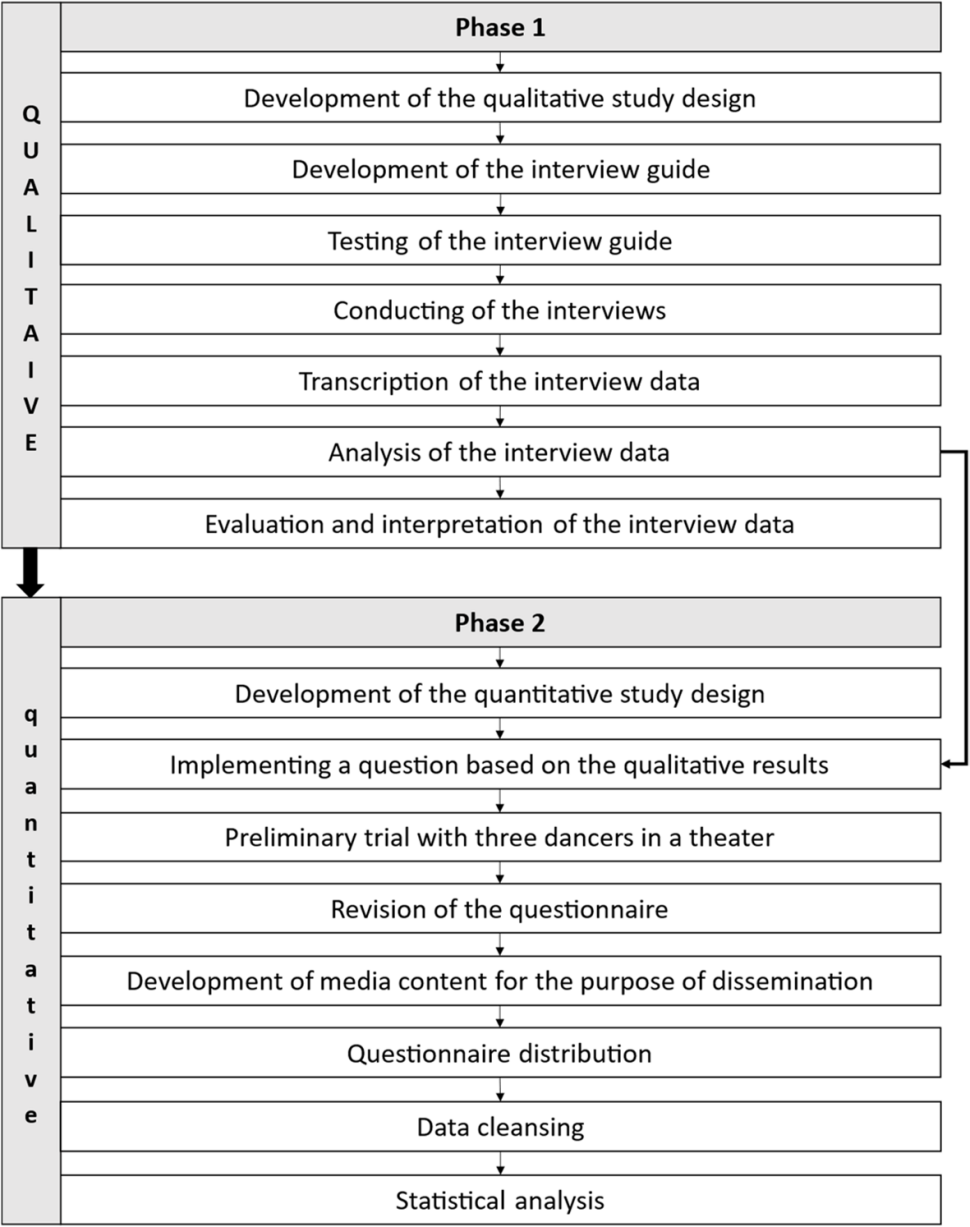
Conception of the exploratory sequential mixed-method-study design (QUAL ➜ quan)

The most appropriate design for this study was determined to be the exploratory sequential mixed-method design for applying 'the house of work ability' framework to professional dance in German theaters. It commences with qualitative phase and subsequently progresses to a quantitative phase informed by the initial findings (QUAL ➜ quan) [28]. The primary qualitative phase provides indispensable insights that inform the development of instruments for the quantitative phase, thereby making this design particularly well-suited to the study of non-specific and intangible phenomena [28]. This mixed-method study design features a dynamic and interactive integration of the methods (Fig. 1) [29].

### Phase 1

A qualitative interview study recruitment was conducted via telephone, email, and social media. The participants were provided with an information sheet in advance, which also included a statement of privacy policy. The interviews were conducted digitally or in person, in either English or German. The interviewer was not known to the interviewees prior to the initial contact to arrange an appointment, but had a background in the field of dance, which facilitated seamless communication of technical dance terminology and created a common level. Participants gave their informed consent at the start of the interview via UniPark Questback GmbH (Cologne, Germany). The interview guide was based on the model of 'the house of work ability' [19] and focused on the fourth floor of work, the work environment, and leadership. Initial inquiries addressed fundamental concepts of work and performance ability alongside their respective influencing factors. Subsequent inquiries explored the congruence between acquired skills and the requirements of professional activities. Furthermore, participants were queried about their sources of knowledge in their present professional context, the competencies they require, and the principles observed in their workplace. The work tasks, the organizational structure of the work, the delineation of the areas of responsibility for each of the involved persons, the influence of hierarchies, the management styles, the modes and structure of communication, and the effects on work and performance were also administered. Finally, inquiries were made regarding the influence of culture and tradition, as well as the desired alterations.

In qualitative research, theoretical saturation represents a criterion for identifying the appropriate sample size [30], which in this instance was determined to be 25 interviews. Subsequent to the transcription process, using the f4x software (dr. dresing & pehl GmbH, Marburg, DE), the interview data underwent qualitative content analysis according to Mayring using MAXQDA (VERBI-Software. Consult. Sozialforschung. GmbH, Berlin, DE) [31]. The categories were formed deductively on the basis of 'the house of work ability' model [19, 20]. More categories of the psychological risk assessment [32] were included in the overarching category work, work environment, work community and leadership. The category system was adapted and expanded using inductively formed categories. A total of 3.005 text segments were assigned from the interviews, which averaged 50.36 min in length (SD ± 14.14 min). This was administered by two individuals for dual coding with the MAXQDA software for to ensure intercoder reliability [33]. The calculation of intercoder reliability was conducted utilizing the MAXQDA software, which was found to be 91.23% with a code overlap of 90%. The statements made by the interviewees in German language were translated in accordance with the recommendations [34, 35].

### Phase 2

An online questionnaire survey was developed and administered. The study population consisted of professional dancers employed in theaters. The online questionnaire comprised 64 items pertaining to sociodemographic data, information related to dance and the abbreviated version of the Work Ability Index (WAI) questionnaire [36]. The sociodemographic information addressed e.g., age, gender, height, weight or ethnic origin. Subsequently, further questioning was conducted regarding the hierarchical structures and the participants' positions within the ensemble. A series of questions were posed pertaining to dance-related subjects, including inquiries about primary dance styles and the duration allocated to various tasks during the workday. The questionnaire employed the shortened version of the WAI, comprising ten questions covering seven different categories [36] and the results of the qualitative interview study were integrated into the questionnaire as a section addressing potential areas for improvement and development [37]. The survey was created and administered using UniPark Questback GmbH (Cologne, Germany). Participants could complete the survey in English or German, and the average completion time was 10–15 min. In order to ensure a broad and diverse range of participants, a multifaceted approach was employed to disseminate information regarding the survey. This included the distribution of posters for theaters, which were equipped with QR codes that directed participants to the survey. Additionally, a newsletter was disseminated through well-known dance institutions, and a video clip was posted on social media platforms over an 18-week period. The data analysis and presentation were conducted using R-Studio (version 4.4.0, 2024–04-24) and Microsoft Excel 2019. A detailed examination of the data was conducted to ascertain their completeness and coherence. For weekly working hours, data points exceeding 12 h per day, 7 times per week, and a cumulative total of 70 h were excluded on the ground of the German regulation [38] specified for performing arts [39], resulting in the removal of a total of 11 data points exclusively for this item. The WAI data were subjected to analysis in accordance with the guidelines of the WAI Network [36], wherein the resulting scores may vary between 7 and 49 points. A score of 7–27 indicates a 'critical' level of work ability, 28–36 indicates a 'moderate' level, 37–43 indicates a 'good level, and 44–49 indicates a 'very good' level of work ability. Body mass index (BMI) was categorized according to the World Health Organization's (WHO) guidelines [40]. To determine the total weekly hours, the hours per day were multiplied by the frequency per week in each respective category, and summed across categories to compute total weekly hours. In order to ascertain the linear influence of sociodemographic variables on the WAI score, multiple nested generalized linear regression models were employed. Table 4 shows which variables were added to the model at each stage (in reverse order). Each model employed robust standard errors to adjust for heteroscedasticity. Test for multicollinearity showed only little correlation for the independent variables. For variables that are quantified in a metric manner, the published coefficients pertain to the slope by which a value increase. Therefore, an incremental increase of one unit of a particular independent variable (e.g., one year for the variable 'age') leads to a change of WAI corresponding to the variable’s coefficient. In the case of non-metric variables or categorical data, the model matrix is employed. Hence, the coefficients indicate the incremental change in the variable in question when it is altered from its baseline value (i.e., the initial level of the variable) to that particular category.

## Results

### Phase 1

A total of 25 individuals, comprising 10 females and 15 males, involved in professional dance were consulted as interview partners. This group included professional dancers (n = 11) and leadership personnel (n = 14), such as ballet masters or theater managers. Additionally, this subgroup encompasses the profession of physiotherapy (n = 2), which is not delineated as a discrete subgroup due to considerations of anonymity. In the following section, quotations will be attributed to the categories of dancers (D) and leaders (L). The mean age of the professional dancers is lower (mean 30.2 years; SD ± 3.9) than that of the leaders (47.1 years; SD ± 9.9). The individuals represented a diverse range of 16 state, municipal, or regional theaters. The ensembles were of varying sizes (mean 34.2 professional dancers; SD ± 21.3). It is also important to note that the ensembles performed a range of dance styles due to the fact that none of the theatres have a repertoire based on a particular dance style.

**Fig. 2:**
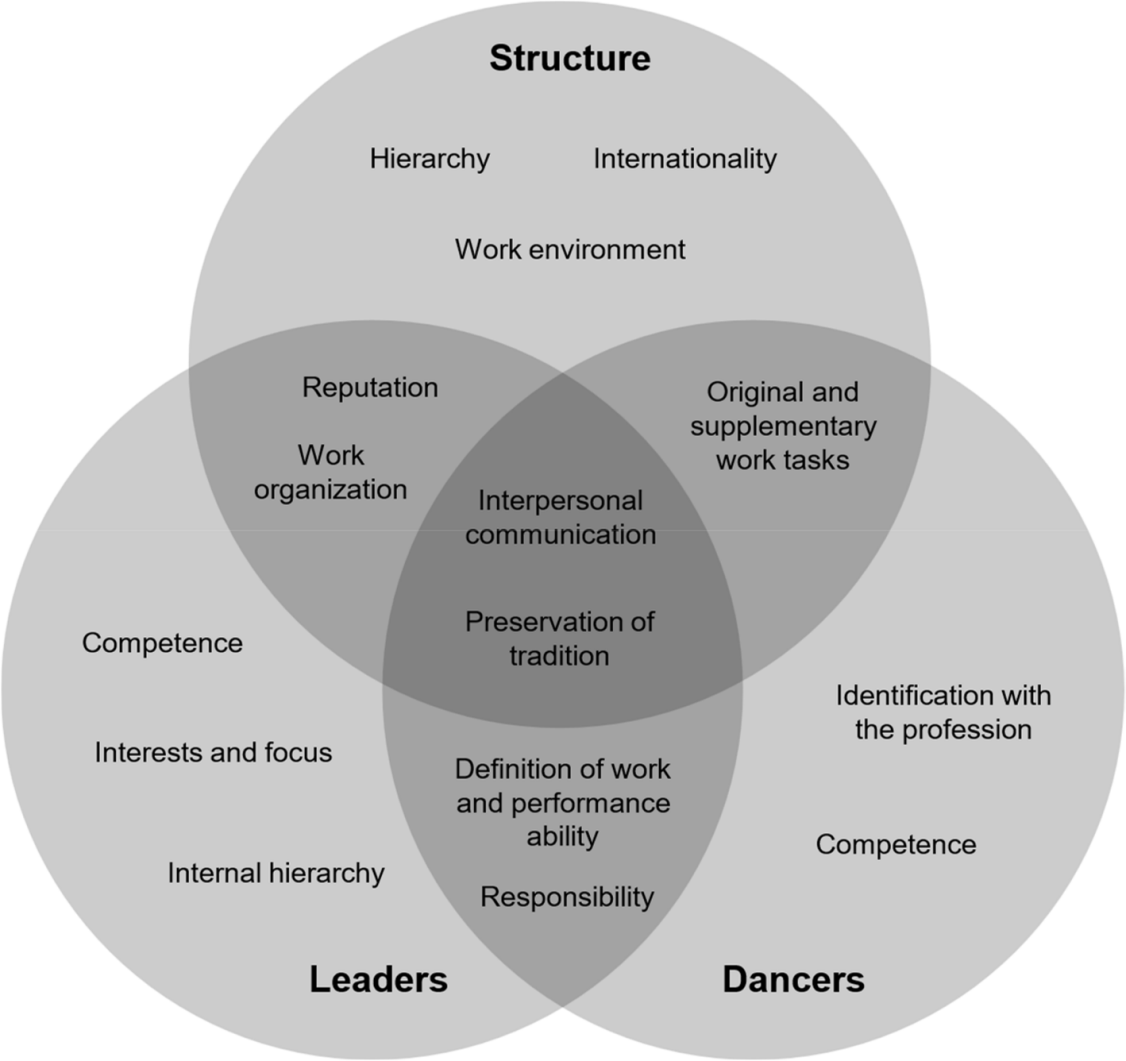
Interplay between structural and individual (leaders/dancers) challenges

Many statements consisted of multiple text segments, frequently classified into disparate categories. This finding indicated the presence of parallels between the interviews, which were recorded initially and are shown in Fig. 2. These results could be sorted into three areas: structure, leaders and dancers which overlap and open up new topics. In particular, communication and the preservation of traditions emerge as common intersections.

Detailed descriptions of the individual categories and associated quotations can be found in Table 1.

**Table 1:** Descriptions of the individual categories structure, leaders and dancers as well as the emerging areas

Structure		
**Hierarchy**	Rigid and actively lived	'Theater is not a democracy.' (L10)
	Barely existent	'It is very loose, to be honest, in my theater. Because we have the usual rank, you know, Principal ballet, Soloist, Demi-Soloist, Coryphée, Corps the ballet^a^. So, we have a lot of ranks. But also, it's very loose because most of the time people who are in Corps de ballet are doing Soloist roles. People who are principals are doing Soloist roles as well sometimes, as like sometimes people who are, for example, like First Soloist or Demi-Soloist are doing Principal roles for quite a long time. So, it is very loose.’ (D6)
	Influence is depending on the position	'It's also, it's depending of your status as a dancer. So, when you are in corps de ballet, maybe you can't really go and say what you need. Not always.’ (D10)
	Disproportionate distribution	'This is the first company I've ever been in where I feel like there are more people above me than there are dancers.’ (D4)
	Power abuse	'And we can kind of say that's abusive. Because you basically don't allow someone to have an opinion or a critical thought. So that's a huge problem for me (laughs).’ (D3)
**Work environment**	Bad working conditions - Less space - Lack of personnel - Missing equipment	'If they always jump in such a small room, where they almost reach the ceiling, it is of course different from having to fill a large room and being able to acquire the condition there at the same time, so to speak.' (L10)
	Good working conditions - Enough space - Physiotherapists - Trainers - Equipment	'We have a very large studio and a nice lounge and checkrooms. They are adequate, I think. Showers. (…) That's all adequate.' (L5)
**Internationality**	Challenges addressing - Communication - Work culture - Life organization	'Our dancers are of course from all over the world. This is an extremely international company. English is our working language, which means they come here to NAME OF THE CITY and the offices don't speak German, of course.' (L2)
**Dancers**		
**Competence**	Wrong knowledge	'Then you realize how much is still going wrong (laughs) and how much false knowledge you have been given.' (D11)
	Missing knowledge in general	'Yes, but training is really marginal.' (D7)
	Missing knowledge about health	'We didn't have enough knowledge about our health, how to take care.' " (D8)
	Missing knowledge about nutrition	'[…] Sometimes they come and say I have no more strength and then you ask: “What did you eat?” “Nothing.” Because they didn't have time. Because they didn't feel like throwing another steak on the pan. […] So that when I need strength now, I just have to eat something.' (L1)
	Long-term view	'Sometimes I think this understanding stops because dancers often don't have the bigger picture.'” (L7)
**Identification with the profession**	Unification of identities	'I think sometimes what happens with us dancers is like I didn´t feel like (…) like a human. Like basically you tag yourself in the name of like I´m a dancer, and then it´s something that you just bring with you. It becomes your identity. While a dancer should be a surplus of what you are, what you do. So, it´s like I´m a human being, therefore I dance. Instead, it becomes like I am a ballet dancer. Point. So, your identity as human is just like going away or is just like everything has to be done for this vocational dancing passion career that you want to have. And then all is focused on that and then they make you believe that you have to be that way, otherwise you´re not going to make it. And for me, that was a big shock.' (D6)
**Leaders**		
**Competence**	Own career in the theater context	'Almost always, when I observe it like this, it's former dancers who then slip into assisting, who then slip into choreography or become ballet directors. Most of the time, that's 90 percent. I'd say they were always dancers of some kind. So, they have the previous training of having been on stage themselves, so to speak.' (L10)
	Additional education	'[…] There are also a few who have actually trained as ballet/dance teachers or ballet masters.' (L11)
	Specific leadership training	'[…] We as leaders, I would call it, are encouraged and it´s offered to us leadership courses, leadership workshops, how to lead a group. How to, yeah, talk with people, discuss, how to conflict management. We do get educated for these kinds of, yeah, so, leadership qualities.' (L14)
	Deficits in dance expertise	'Now we've had a new artistic director […], who comes from the acting department. Unfortunately, he is completely ignorant and completely uninformed about dance and doesn't understand it at all.' (L2)
	Deficits in management expertise	'In my career right now, I haven't seen ANYONE actually knowing what being in a leadership position means, and they lack completely in mana/ management skills, working with the people and motivating people.' (D6)
**Interest and focus**	Interest	'I think at the moment it's a problem that we've addressed, […] but it's quantity rather than quality.' (D1)
	Vision	'I would say the Intendant creates a very clear kind of aesthetic preference for the theater. And then the ballet oftentimes functions somewhat independently.' (D2)
	Finances	'We don't have the money for everything.' (L3)
**Internal hierarchy**	Procedures	'But then, if not with him, then it goes to the director in the NAME OF THE THEATER. And I haven't had to refer it yet, but if it doesn't go any further, then it has to go to the mayor NAME OF THE CITY. That would then be the official channel for me, so to speak.' (D1)
	Mutual understanding	'We really had to fight to be out by ten o'clock and the management simply didn't understand. An understanding of how we had to work.' (L2)
	Authorities	'So, our director has the ABSOLUTE say and he is a control freak.' (L7)
**Structure x Dancers**		
**Original work tasks**	- Classical / non-classical regular training - Rehearsals - Performances - Special work tasks	'For instance, like we in the company that I'm in, we train in ballet most of the time, I would say like well over 50, 60 percent of the time we're doing ballet, maybe even more and a lot of the work that we perform is not necessarily balletic. And so, yeah, there's a strong tradition in like Western concert dance of like using ballet as the as the cover all training.' (D2)
**Supplementary work tasks**	- Lifestyle adjustment - Performance adjustment - Self-responsibility - Staying fit - Additional training - Rehearsal preparation	'They definitely need to make sure that they get enough sleep, that they eat healthily. Sleep, a healthy diet, um, and then everything that helps them stay fit. Um. Like massages, sauna. Massage, sauna. Otherwise, lots of fresh air. Maybe some people underestimate the fact that this also leads to a healthy body.' (L3)
**Structure x Leaders**		
**Reputation**	Within the theater	'I have to tell you honestly, if you look at the departments in the house. What a value dance has, purely financially at such a small house compared to music theater. […] They are soloists with us, so they have a solo contract and that brings them up to the minimum fee, which has now increased. We are very, very happy about that, although our company can't afford it either. And we're afraid that the dance division will be wound up because of it. But when you see how an orchestral musician starts, with what salary, let's say a monthly salary, which also starts early. But a dancer?' (L2)
	Within the city	'I think that's suboptimal. Not just for dance, but for this machine in general, this city theater machine.' (L5)
**Work organization**	- Training management by leaders - Lack of participation - Overloading - Brief announcements - Coordination challenges between departments - Absence of effectiveness	'I wish rehearsals could be more compact and I feel I would be a lot more productive. I would be a lot more motivated. And I feel working six days a week is not good on our bodies. I feel it's very strenuous and I do really feel it. A lot of normal companies, you know, were trialing this four-day workweek. Four days, and you sit behind a desk?' (D4)
**Dancers x Leaders**		
**Definition of work and performance ability**	Work ability - As a continuum - Connected to work attitude - Sufficient recovery time and an adequate warm-up needed - Physical and mental health serve as foundation	'Because (..) oftentimes we are not performing at 100 percent, not necessarily performing on stage, but I mean, just like performing our work in the day-to-day basis at 100 percent. Um, I think there's always like a gradient of (laughs) (…) Yeah, energy or injury or mental, emotional, spiritual existence.' (D2)
		'But I never skip work, for example, because I don´t feel like going or I´m tired […] so, you know that you have to do […] and sometimes we also have to push ourselves […] And we always try to do like our best and even if you have pain, you want to dance, dance, dance. Then I remember I dance until I couldn´t walk, but I danced.' (D8)
	Performance ability - Equal to work ability - High degree of individuality - Dependent on general health, training status, sense of security, time within a season, stress level, female cycle - Has its limits	'It's the same. They're giving eight hours a day in the studio, 150 percent to the choreographer and then the expectation is the same then when they're performing.' (L14)
		'Of course, we always try to do our best. Always 100%. But we are also not robots.' (D8)
**Responsibility**	Delegation of responsibility to dancers	'Take a bit of personal responsibility. (…) And (…) yes, that they don't complicate the work processes with personal sensitivities (laughs).' (L7)
	Delegation of responsibility to leaders	'That you're not responsible for getting your body in shape for the next production, but that you have one or two weeks of adoption time between two productions where you know that they are physically very different.' (D11)
**Structure x Dancers x Leaders**		
**Interpersonal communication**	Transparent communication	'So, from that point of view, it's good and communication with the dancers is good anyway. Of course, when new dancers come, a bit of trust-building is important, but otherwise they spend a lot of time with us and the more they know us, the more they tell us and the more you get to hear.' (L13)
	Problematic communication	'Yeah, I think in general we are having issues with communication. Yeah, I would say (laughs). But it goes/ It´s again, it´s very different to each person. maybe some people have less problems to communicate with the/ With our stuff and some dancers have more problems to communicate with them.' (D10)
**Preservation of tradition**	Personal attitudes	'Our director or the ballet masters, they all believe like whatever used to be before, it's what's right.' (D10)
	Contrast to increasing requirements	'So, I think, for example, in the seventies, eighties, maybe all these great choreographers, they didn't pay so much attention to that, but also because what they were asking their dancers wasn't as demanding as what it is now.' (D5)

^a^ A hierarchy within a professional dance ensemble is described in ascending order from pre-professional dancers (= Apprentice/Élève), group dancers (= Corps de ballet), the preliminary stage of soloists (= Demi-Soloists), Soloists (= Soloists) and the main roles or faces of the ensemble (= First Soloist/Principal) [3, 41]

### Enhancement potentials

Certainty in planning received considerable attention encompassing aspects such as time, work content, private life, and more efficient work organization. Another frequently discussed topic is the enhancement of the work environment, addressed with greater frequency by leaders. The following factors were identified: the size and availability of training and rehearsal spaces, the installation of sprung floors, the reliability of cleanliness, the adequacy of sanitary facilities (e.g., showers), the provision of a cafeteria, and the availability of temperature regulation options (air conditioning), regeneration facilities or the need for changing locations. Additionally, professional development and workshop opportunities, encompassing both job-specific and non-job-related content, has been raised with greater frequency by the leaders. The aforementioned tendency also pertains to the reduction in the number of productions per year to prioritize quality over quantity and the expansion of the personnel complement. Two areas that were addressed exclusively by the leaders were improvement of communication in the theater and participation of the professional dancers in the programming and scheduling of performances:

'[…] I would even like to see a conference between directors and dancers to shed light on the other side. What it's like when dancers/ And really give case studies of how they sometimes exploit dancers, the situation they're in' (L7).

Furthermore, there are also topics that were more frequently discussed by the professional dancers. These included the enhancement of recovery, regeneration, and transition phases such as adaptation to new movement patterns with highly diverse choreographic demands:

'Even if I still and will never stop fighting to make this even more important, because I believe that in every other competitive sport, you work precisely in these mechanisms and always make sure that after a competition, i.e., after a peak performance, a break phase must be included so that the body can regain its energy and, yes, initiate all the regeneration processes' (D11).

The expansion of training variability and the reduction of reliance on classical training were mentioned, as well as the incorporation of a diverse array of athletic training, yoga, pilates, and other dance styles, facilitated by a variety of trainers. The issue of enhancing leadership abilities was also identified:

'(14) I think, like I talked about earlier. A huge issue for me is people that aren't qualified in their positions that run a company or run people sometimes into the ground damaging their physicality, their body, their mental health. All because of art. That I think that needs to be someone that is either in the art world, that is studied about management, about how to run a company, a ballet company, or a collaboration between two people equally all the time. […] Just because you do it doesn't mean that you are qualified to do that for people in that profession.' (D4).

Both subgroups addressed the issue of expanding support and care for professional dancers in comparison to high-performance athletes:

'In my opinion, that we do not have the treatment, we do not have the assistance that an athlete has. If you compare dancers with soccer players or rugby players or swimmers, oh my god, tennis players, oh my god, you know. We deserve it that way they focus on that person and that athlete and/ What is necessary to achieve the best of that person. It's hard to compare with dancers, because we don't get any of that, starting with the money' (D9).

The provision of physiotherapeutic support or cooperation with physiotherapy practices is regarded as the foremost priority. Additionally, psychological support is frequently cited by the professional dancers. Furthermore, there are quotes on nutritional counsel, training guidance provided by qualified professionals such as sport scientists, and medical care uniquely designed for professional dancers. The provision of training equipment (e.g., training mats, foam rollers, strength training equipment, Gyrotonic machines) and the improvement of contract conditions (payment and contract duration) were mentioned less frequently as well as the development of dance-specific rehabilitation programs after injury or illness:

'Everybody is struggling coming back because nobody is there to support you, to help you' (D10).

The areas identified were incorporated into the response options of the quantitative survey. The results of the ranking of these options can be found in Fig. 4.

### Phase 2

The survey was completed by n=75 professional dancers, whose characteristics are presented in Table 2. Most participants were between 20 and 29 years old (49.4%), a higher proportion of female participants compared to male participants, and a larger proportion of individuals with a healthy weight BMI. Additionally, the sample showed high international diversity, with participants from over ten different national backgrounds, with increased participation by individuals from the corps de ballet and a preponderance of classical ballet and contemporary styles.

**Table 2:** Participant characteristics (Phase 2)

**Age**		
Mean ± SD: 28.76 ± 7.74 years		
18–29: 51 (49.4%)	30–39: 18 (30.8%)	≥ 40: 6 (19.8%)
**Gender**		
Female: 47 (62.7%)	Male: 27 (36.0%)	Non-binary: 1 (1.3%)
BMI*		
< 18.5: 10 (13.3%)	18.5–24.9: 63 (84.0%)	25–29.9: 1 (1.4%)
**Ethnic Origin**		
German: 16 (21.3%)	Swiss: 4 (5.3%)	Italian: 18 (24.0%)
Austrian: 1 (1.3%)	French: 1 (1.3%)	British: 3 (4.0%)
Belgian: 1 (1.3%)	Spanish: 3 (4.0%)	American: 3 (4.0%)
Canadian: 4 (5.3%)	Other: 21 (28.0%)	
Position**		
Apprentice/Élève: 2 (6.9%)	Corps de Ballet: 13 (44.8%)	Demi Soloist: 5 (17.2%)
Soloist: 6 (20.7%)	First Soloist/Principal: 3 (10.3%)	
**Size of the ensemble**		
0–19: 39 (52%)	20–39: 18 (24.0%)	40–59: 5 (6.7%)
> 60: 13 (17.3%)		
**Primary dance style**		
Classical Ballet: 34 (45.3%)	Contemporary: 30 (40.0%)	Jazz Dance: 3 (4.0%)
Latin/Ballroom: 2 (2.7%)	Modern Dance: 3 (4.0%)	Hip-Hop: 1 (1.3%)
Other: 2 (2.7%)		
Number of weekly working hours***		
< 20 h: 1 (1.2%)	20–39 h: 10 (15.6%)	> 40 h: 53 (82.8%)
**Number of workplaces**		
1: 45 (60%)	2: 25 (33.3%)	> 3: 5 (6.6%)

All data are presented as n (%)

^*^The provision of information regarding weight and height was voluntary. One participant did not submit any data in this category

^**^The specification of the position within the ensemble was only possible under the condition that general hierarchical structure was specified. The final *n*=29 is displayed for this particular item

^***^For total weekly hours, values exceeding 12 h/day, 7 days/week, or 70 h/week were deemed implausible and excluded, removing 11 data points and a final *n*=64 for this item

**Fig. 3:**
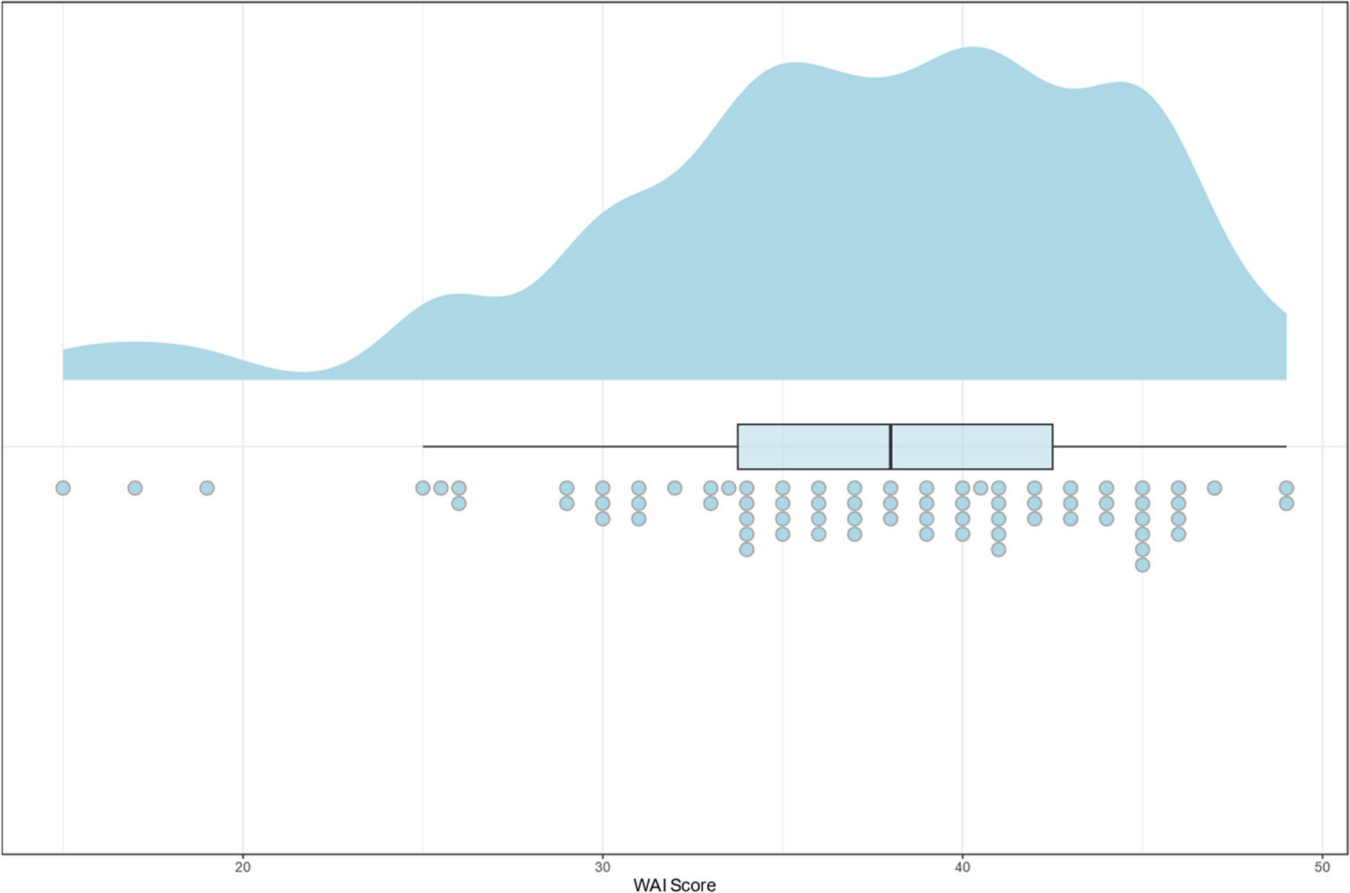
Raincloud plot of work ability index in the sample (N = 75)

The mean WAI was 37.18 (SD ± 7.18) and the work ability differs considerably with ranging scores from 15.0 to 49.0. The majority of the professional dancers indicated moderate to good work ability (Fig. 3).

Notably, the disparities in ethnic background, hierarchical position within the ensemble, and the decline of WAI with an increase in ensemble size are particularly noteworthy in relation to the WAI values across the distinct categories shown in Table 3. The WAI was found to be greater among professional dancers that did not adhere to the principles of a hierarchy (38.57; SD ± 8.74) than in theaters with a structured hierarchy (35.53; SD ± 5.28).

**Table 3:** Mean WAI across sociodemographic and dance-related characteristics (Phase 2)

**Age group (years)**		
18–29: 36.38 (SD ± 7.38)	30–39: 38.4 (SD ± 6.8)	≥ 40: 40.3 (SD ± 6.76)
**Gender**		
Female: 36.75 (SD ± 8.12)	Male: 37.96 (SD ± 5.38)	Non-binary: 37.0
**BMI**		
< 18.5: 34.40 (SD ± 10.89)	18.5–24.9: 37.67 (SD ± 6.54)	25–29.9: 37.0
**Ethnic Origin**		
German: 36.81 (SD ± 7.92)	Swiss: 36.87 (SD ± 9.54)	Italian: 36.94 (SD ± 5.2)
Austrian: 46.0	French: 29.0	British: 37.66 (SD ± 5.5)
Belgian: 43.0	Spanish: 35.0 (SD ± 8.18)	American: 36.0 (SD ± 1.0)
Canadian: 44.25 (SD ± 2.98)	Other: 36.52 (SD ± 8.67)	
**Position**		
Apprentice/Élève: 45.0 (SD ± 5.65)	Corps de Ballet: 37.34 (SD ± 6.35)	Demi Soloist: 25.8 (SD ± 12.27)
Soloist: 35.75 (SD ± 6.7)	First Soloist/Principal: 39.33 (SD ± 2.88)	
**Size of the ensemble**		
0–19: 38.68 (SD ± 5.87)	20–39: 37.08 (SD ± 8.04)	40–59: 36.8 (SD ± 10.2)
> 60: 33.15 (SD ± 7.41)		
**Primary dance style**		
Classical Ballet: 35.98 (SD ± 7.74)	Contemporary: 37.88 (SD ± 6.51)	Jazz Dance: 39.5 (SD ± 12.37)
Latin/Ballroom: 42.5 (SD ± 4.94)	Modern Dance: 40.66 (SD ± 5.5)	Hip-Hop: 37.0
Other: 33.5 (SD ± 3.53)		

All data are presented as the mean WAI with standard deviations in brackets when greater than n = 1

Factors that impacted the individual WAI were tested by several nested generalized linear regressions, as illustrated in Table 4. The constant of model (3) is used to describe the estimated value of the dependent variable at a value of zero for all predictors.

**Table 4:** Regression model for n=75 professional dancers

	Dependent variable: WAI						
	(1)		(2)		(3)		
	*Coef*	*SE*	*Coef*	*SE*	*Coef*		*SE*
Age	0.071	(0.168)	0.224	(0.168)			
Gender: female	0.668	(1.881)	0.462	(1.836)	0.677		(1.714)
Gender: non-binary	-3.057	(5.904)	-1.784	(6.371)	-1.417		(6.207)
BMI 18.5- 24.9 ‘healthy weight’	0.382	(2.544)					
BMI 25.0- 29.9 ‘overweight’	1.658	(7.400)					
Ethnic origin: non-German	0.032	(2.196)	0.534	(2.159)			
Position: Apprentice/Elevé					6.491	(4.852)	
Position: Corps de ballet	1.889	(3.294)	-0.544	(2.744)	-1.154	(2.519)	
Position: Demi soloist	-12.478***	**(3.918)**	-14.258***	**(3.861)**	-18.161***	**(3.497)**	
Position: Soloist	-0.748	(3.225)	-2.071	(3.154)	-2.936	(2.705)	
Position: First soloist/ Principal	2.505	(4.556)	1.686	(4.461)	0.962	(4.330)	
Size of ensemble	-0.037	(0.052)					
Primary dance style: Contemporary	1.668	(2.348)	0.167	(2.227)	-0.180	(1.942)	
Primary dance style: Jazz	-2.797	(5.195)	-2.851	(4.920)	-0.792	(4.155)	
Primary dance style: Other	-2.096	(3.954)	-1.005	(3.204)	-1.091	(3.078)	
Number of weekly working hours	0.056	(0.084)	0.023	(0.088)			
Number of workplaces	-3.437***	**(1.004)**					
Actively dancing	0.120	(0.097)	0.117	(0.106)	0.004		(0.043)
(Co-)Choreographing	0.178	(0.162)	0.140	(0.175)			
Dance-related teaching	0.236*	(0.123)	0.153	(0.131)	0.037		(0.078)
Non-dance-related teaching	-0.051	(0.167)	-0.103	(0.181)			
Constant	26.046*	(13.506)	19.274	(13.395)	37.919***	**(4.467**)	
Observations	60		61		68		
Log Likelihood	-175.290		-186.773		-211.525		
Akaike Inf. Crit	392.580		407.546		449.051		
Adj.${R}^{2}$	0.41		0.27		0.28		

^*^p < 0.1, **p < 0.05, ***p < 0.01; bold p-values are considered as significant;

Displayed are the coefficients estimates and their respective standard errors (in brackets)

**Fig. 4:**
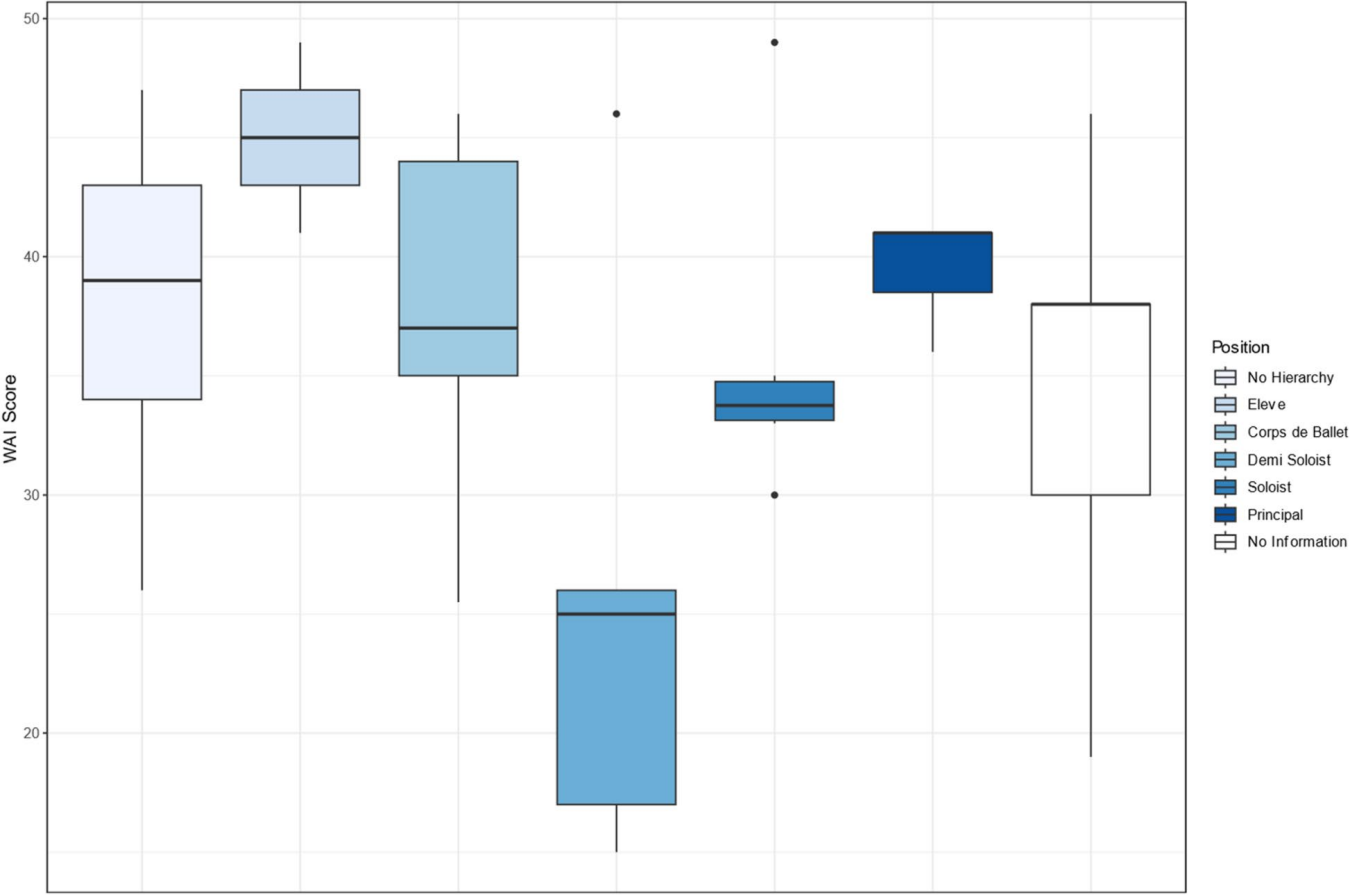
Distribution of Work Ability Index scores across different ensemble positions (no information = no hierarchical structure within the ensemble)

The number of workplaces (ß = -3.437, SE = 1.004, p < 0.01) was found to be a significant factor in model (1). Nevertheless, the most notable observation pertains to the demi soloists, who are associated with lower predicted WAI values across all three models compared to the other positions shown in Fig. 4 (Model (1): ß = -12.478, SE = 3.918, *p* < 0.01; Model (2): ß = -14.258, SE = 3.861, *p* < 0.01; Model (3): ß = -18.161, SE = 3.497, *p* < 0.01).

Given the highlighted disparities observed in the initial analysis and the previous qualitative findings, a more explicit analysis was conducted. A significant difference between the WAI of demi-soloists and the other positions (Kruskal–Wallis chi-squared = 5.2141, df = 1, *p*-value = 0.0224) as well as between the ensemble size and WAI (Kruskal–Wallis chi-squared = 5.3075, df = 1, *p*-value = 0.02123) was found. Additionally, there is a notable difference between existing hierarchical structures and the WAI (t = -1.7777, df = 52.038, *p*-value = 0.0813).

**Fig. 5:**
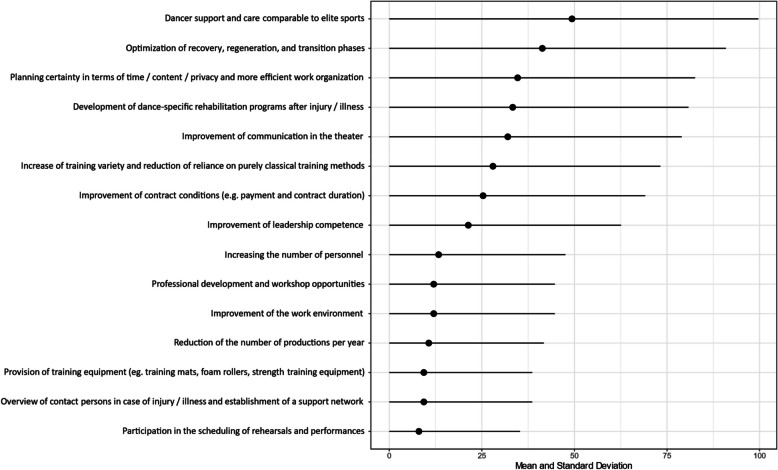
Ranking of changes perceived as important by n=75 professional dancers at the theater (multiple answers possible)

A thorough investigation into the potential areas for improvement and development was conducted in addition to the administration of the WAI survey. In the context of the ranking of changes perceived as an important change, the professional dancers were permitted to report more than one potential change (Fig. 5). The category *Dancer support and care comparable to elite sports* permitted multiple specifications to be indicated. The most chosen specification was *Medical care specifically tailored to dance* by 29 professional dancers, closely followed by *Physiotherapy services and/or collaborations with physiotherapy practices* which was indicated by 28 professional dancers. The specifications *Training supervision/Sports scientists* and *Psychological support* were equally often selected by eleven professional dancers. Two professional dancers chose the specification *Other*. In the category of *Improvements in the work environment* respondents were permitted to indicate more than one specific area of improvement. Of the nine professional dancers who chose this category, eight supported the specification *Size and availability of training/rehearsal spaces*. Six professional dancers each indicted their desire for the specification *Regeneration facilities*, *Installation of sprung floors* and *Other*. Further two professional dancers reported their desire for a change in the specification *Reliable cleanliness*. The specifications *Adequate sanitary facilities*, *Reduced need for changing locations* and *Cafeteria* were only selected by one participant each.

## Discussion

The study aimed to identify challenges and opportunities within the work ability framework for implementing measures in theaters to maintain work ability and enhance the performance ability of professional dancers. This investigation was undertaken with the understanding that professional dancers engage in elite sport in an occupational setting.

The majority of the participants exhibited moderate to good WAI scores. In comparison to the general working population [42, 43], which demonstrates WAI in the good range, the professional dancers display lower WAI scores. These results can be explained by the high level of occupational physical activity, given that there are parallels in the literature with lower WAI when shown minor or substantial increases in physical activity [21]. In the present study, Apprentices/Élèves (6.9%) exhibited the highest WAI. This phenomenon may be attributed to the comparatively less strenuous and less diverse nature of the workloads experienced by these individuals [44] in comparison to professional dancers who are engaged in work after theater timetables [3]. It indicates the necessity for disparate analyses for professional and pre-professional dancers, given their distinct career stages and work demands [45, 46]. In contrast, 9.3% of the professional dancers demonstrated critical WAI scores, indicating the presence of a vulnerable subgroup. The WAI scores were found to be significantly lower among Demi-Soloists, who bear dual responsibilities. They often face additional physical and mental demands as they are required to perform both in the Corps de ballet and as Soloists. Studies show that WAI is influenced by high physical and mental work demands [47]. More research is needed to investigate the role of Demi-Soloists, as the complexities of their responsibilities may lead to distinct work ability as well as performance ability issues compared to other positions in the ensemble. This would also be necessary to develop measures specifically tailored to this target group.

Especially when working in a theater, as indicated by the qualitative results, the issue of work-life balance is a persistent challenge for professional dancers. Participants find it difficult to maintain a private life while fulfilling the demands of their professional roles. This imbalance, coupled with restricted prospects for further education, aligns with and is additionally reinforced by previous research, which underscores the broader structural challenges confronting professional dancers, including job insecurity, inadequate payment, and the inability to pursue long-term career pathways [11, 12, 48]. Given that the majority of participants were within the typical age range for professional dancers, these concerns are further compounded by the relatively short career span in the field of dance [49]. It is therefore imperative to emphasize the importance of job-specific education and career transition programs in order to enhance the employability of professional dancers outside of the dance industry. This is further evidenced by the expressed desire for professional development and workshop opportunities (10th place). As shown in the literature, a longitudinal investigation of the relationship between opportunities for development, autonomy in scheduling, and the quality of leadership found a positive effect on WAI [50].

Presently, there is a limited degree of autonomy, particularly in the realm of work organization. The hierarchical structures in place necessitate the central role of management. It becomes evident that, despite the presence of former professional dancers in these interface positions, a structural dichotomy between artistic and administrative processes emerges [12]. This can have an impact on the dance sector's ability to function effectively and can affect the professional dancer’s work ability and resulting performance ability. Moreover, the desire to enhance leadership skills was identified as the eighth most prevalent improvement sought by respondents in the survey. The responsibilities in question are not limited to a single strategic function; rather, they encompass a broad spectrum that is also present in the other improvements deemed necessary including the planning of the regeneration, recovery, and transition phases (2nd place), the planning of certainty and efficient work organization (3rd place), and the decision-making process regarding the variety of training (6th place).

It is noteworthy that professional dancers indicate that improved workload management and increased involvement in planning decisions could have a positive impact on work ability and enhance their performance ability. This is further intensified by the literature about insufficient rest and recovery time [51, 52]. In this context, interview results also describe the organization of work, including simultaneous training planning, which is carried out by the leaders and is partly dependent on internal hierarchies at management levels. Thus, the organization of work is predominantly driven by artistic interests [12], with limited consideration given to the principles and methodologies of sport science. Additionally, the inefficiency of theater operations reported, contributes further to the overwork and exhaustion of professional dancers. Matussi et al. [53] highlight the significance of periodization and rest, which are common practices in elite sport but are often overlooked in professional dance. This suggests a need for theaters to adopt scientific, evidence-based principles for workload management in order to prevent overloading and ensure sufficient recovery time, in a manner analogous to that employed in professional sports [54, 55]. This approach would be mutually beneficial, as recent studies indicate that approximately 75% of all injuries are attributable to overuse injuries [53, 56], which can lead to a reduced ability or complete inability to work.

Furthermore, the interview results indicate that the general planning is often characterized by a short-term perspective and spontaneous alterations. Additionally, the work tasks, such as rehearsals, can be considerably time-consuming and ineffective, exhausting professional dancers physical and mental resources. Moreover, the original and supplementary work tasks were identified as a component of the professional dancer's responsibilities beyond the scope of their contractual obligations. This commitment is primarily motivated by their profound dedication to the profession. It is essential to supervise the entirety of training that occurs outside of standard theater working hours to prevent excessive physical exertion, a factor that according to literature contributes to injuries [57]. In addition, the incorporation of supplementary tasks is necessary to preserve work ability and avoid overload beyond the parameters of the contractual obligations, because research indicates that injury prevention can be conceptualized as a dynamic balance between load preparation and load management [58]. With regard to the responsibility for planning the working time of professional dancers, and thus the loading and unloading phases, it is vital that the leaders have a fundamental understanding of training principles [55]. Despite the variability inherent in artistic processes, it is imperative to establish long-term structures that provide regulated working hours and intentions, with the objective of preserving the professional dancers' physical and mental resources. Furthermore, a high level of awareness of the professional dancers' needs and the implementation of sustainable informed and evidence-based decisions in these areas could have a positive impact on professional dancers work ability and enhance performance ability.

With regard to the variety of training within the work demands of the professional dancers, it is necessary to recognize that the qualitative results show a growing interest in incorporating more diverse training regimens and enhanced warm-ups into classic dance training. According to literature, these modifications aim to better prepare professional dancers for the demanding physical demands at the workplace, rather than relying on a single, standardized warm-up routine that is largely based on tradition and lacks scientific evidence [59, 60]. While traditional classical dance training should not be entirely replaced, findings suggest that theaters may benefit from the integration of regular supervised athletic training, adapted to the requirements of the skills demanded by the piece at the time, into the daily training routine. In contrast to the current approach of imposing these demands on an individual basis, this strategy offers a more holistic and integrated approach complementing and enhancing the classical training component. Additionally, it would align with current stylistic dance demands in theaters, which have evolved beyond the exclusive presentation of classical ballet dance [61, 62].

Another potential improvement that has been identified is the reduction of the number of productions per year. The explanation could be the structure of the subscription system in Germany, which requires the production of a significant number of new works each year, which places considerable strain on professional dancers [48]. One potential solution to this pressure is to transition towards a model with a smaller number of higher-quality productions. Such a system would also allow for the implementation of an 'off-season' or transition phase, as is common in elite sport. This would provide professional dancers the opportunity to recuperate from their previous season, rest, and transition into a 'pre-season', during which the fundamental elements for performance are established. These include endurance and strength training, which serve as the foundation for more individualized training [63, 64]. An increase in staffing could also serve to reduce competition and distribute the workload more evenly; however, budgetary constraints in the cultural sector present a significant barrier to this solution. Despite substantial cultural funding, theaters frequently encounter difficulties in maintaining economic viability, often failing to generate sufficient revenue to cover costs [65]. This phenomenon is further substantiated by the outcomes of the conducted interviews, which indicate that the prevailing work environment is not appropriate for e.g., training, rehearsals or regeneration measures. Specifically, the dimensions of the rehearsal space are inadequate, as the available rooms are too small and do not correspond to the dimensions of the stage, thereby failing to provide a realistic training stimulus during rehearsals. Since infrastructural problems, particularly those related to physical facilities, cannot be readily addressed due to complex interdependencies, attention turns to external factors that contribute to maintaining work ability and enhancing performance ability.

Prior research has indicated that pronounced hierarchies can impede work efficiency and increase the probability of power abuse [66]. This impact is evident both in the significant effects on WAI observed in larger, more hierarchically structured ensembles and in the interview data documenting power abuse. The findings additionally reveal that the hierarchical structures that are still inherent to theatrical organizations foster a culture in which professional dancers internalize the necessity to meet exceptionally high standards. Professional dancers appear to feel compelled to perform despite injury, driven by a fear of missing opportunities, particularly within the competitive and hierarchical structure of dance ensembles [67]. Consequently, this can result in detrimental self-perception and, in some cases, abusive power dynamics due to asymmetric structures, as highlighted by Schmidt [12, 22, 48]. These dynamics are inextricably linked to long-term job insecurity and the pressure to conform to management expectations. Furthermore, such structures can impede effective communication, which in turn diminishes participation [68]. Interpersonal communication is one of two challenges arising from the intersection of the three primary domains of leaders, professional dancers, and structure. These findings highlight a significant gap between theoretical ideals and practical reality in workplace communication. The literature emphasizes that effective communication, particularly direct information sharing with employees and establishing trust-based relationships, constitutes a fundamental prerequisite for successful workplaces, where employees are heard and their voices actively shape workplace design to ensure social sustainability [69]. The study's findings directly contradict these theoretical foundations. The absence of psychological safety in communication hinders the process of doing, and understanding that research identifies as essential for transformative and behavioral learning [70–72]. In light of the identified communication gaps, organizations are advised to implement structured mechanisms that ensure psychological safety in workplace communication. Such mechanisms should include anonymous feedback systems and protected dialogue formats that enable transparent information sharing without fear of retaliation.

The communication gaps identified are further compounded by cultural traditions that hinder open dialogue concerning workplace challenges, with the preservation of such traditions constituting the second challenge. This area is therefore an essential factor for bringing about change, because studies show that prolonged exposure to adverse working conditions has an impact on reducing WAI [50]. The preservation of tradition is reflected in various areas, such as structural working methods, training regimes, and dealing with pain. Especially, the ethics of ballet place emphasis on resilience and endurance. Professional dancers who demonstrate the ability to work through discomfort, even when it risks long-term health, are held in high regard [73]. This pervasive attitude that physical strain is a normal part of the job, as discussed by McEwen and Young [74], serves to reinforce the tendency to continue performing while in pain. In addition, professional dancers were found to have incorrect knowledge in general and a lack of knowledge in the areas of nutrition and health. According to literature, they are also inculcated with the notion that persevering through discomfort is essential for achieving success, thereby fostering a culture where overuse injuries are perceived as indications of dedication [73]. This reflects the attitude, that professional dancers feel compelled to perform despite injury due to the fear of missing opportunities, particularly within the competitive and hierarchical structure of dance ensembles [67]. Building on this, the results of the interviews still demonstrate the professional dancers' strong identification with their profession, reflecting the cultural characteristics of their attitudes. Nevertheless, professional dancers also have expressed both reflections on and criticism of this phenomenon. In this context, it is relevant that psychological support was a high ranked item of desired improvements, and should not be overlooked in favor of improving physical conditions to prevent mental illnesses, including burnout and chronic fatigue, that are currently inherent [75]. Therefore, the provision of psychological support would not only be important for the current professional dancers at the theatres, but also a change in the traditional culture of pain [74]. There is a need for the establishment of a new culture that respects tradition while also prioritizing progress. This approach would focus on the biopsychosocial health of professional dancers, serving as an essential foundation for their work ability to enhance performance ability. Nevertheless, the qualitative results show that the distinction between work and performance ability was not clearly delineated, particularly between the leaders and the professional dancers. The operationalization of work ability as mere workplace attendance may result in misguided positive assessments in performance evaluation contexts. Interpretations of this kind are inadequate in accounting for the significant discrepancy between physical presence and a professional dancer's actual performative capacity. If this is taken as a foundation for how the support of professional dancers is given in the theater, a potential step forward could involve a unified definition of work ability and performance ability based on common definitions in the sport and occupational sciences.

In light of the scientific findings from the study, the following proposal for a novel definition is hereby presented. Work ability should be defined as the multidimensional capacity of a professional dancer to meet occupational demands. This capacity encompasses physical, psychological, and social components required for unmodified participation in class, rehearsal, and performance activities. Performance ability is defined as the integrated, multidimensional capacity of a professional dancer to not only fulfill professional requirements, but to exceed them at an optimal performance level through the execution of choreographic requirements with technical precision, artistic expression, and physiological efficiency. This concept extends beyond fundamental work abilities, encompassing a synergistic integration of sport-specific and dance-specific competencies that enable the maintenance of optimal biomechanical integrity throughout the duration of performance. These competencies are supported by individual characteristics and structural framework conditions, culminating in the delivery of exceptional artistic and athletic performance. In summary, performance ability encompasses more than mere work ability, signifying the capacity to optimize performance execution, exhibit adaptability, and undergo continuous development within the professional domain while maintaining technical and physiological excellence.

**Fig. 6:**
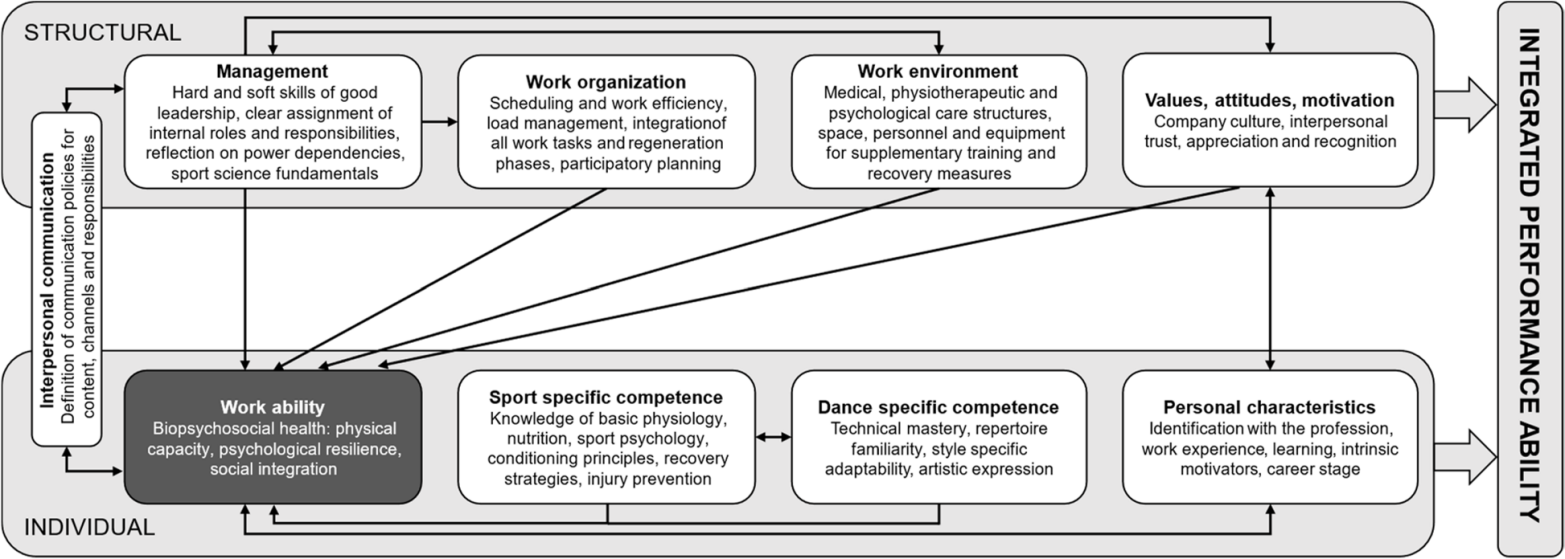
Model of integrated performance ability in professional dance

The model of Integrated Performance Ability in professional dance proposed in Fig. 6 offers a multidimensional framework for understanding performance ability in the context of professional dance in a theater. This model draws parallels with established frameworks in occupational health and performance psychology, including the Job Demands-Resources model [76, 77], House of Work Ability [19], Salutogenesis [78], or biopsychosocial approaches [79]. While the extant models provide valuable insights, a context-specific framework is necessary to capture the unique dynamics of professional dance environments. It conceptualizes performance ability not as an isolated individual achievement, but as the result of continuous interactions between individual capacities and structural conditions. The model reframes work ability as a context-sensitive enabling condition that supports task engagement and adaptability, while performance ability is understood as a systemic and co-produced outcome. This integrated view challenges narrow, individualistic interpretations of performance and suggests instead that sustained high-level functioning in professional dance is contingent upon supportive structures, reciprocal adaptation, and alignment between individuals and institutions. As such, the model provides a basis for both diagnostic assessment and strategic development, relevant for health promotion, career longevity, and institutional learning in the professional dance sector. This framework would be most effective provided that institutions are willing to engage in both preservation of traditional practices and adaptation to emerging needs.

## Limitations

Voluntary recruitment method in the interview study is associated with selection bias. This methodological limitation suggests that participants may exhibit greater topic engagement and interest compared to the broader population of leaders and dancers in German theaters. Nevertheless, rather than seeking generalization, this qualitative component aims for transferability, which allows for a more nuanced understanding of how these findings might apply to other contexts. Readers are encouraged to assess the potential applicability of the information based on the detailed contextual information and dense descriptions provided [33]. Although a register of the professional dancer population exists through employment records at theater institutions, these were not accessible due to data protection regulations. Consequently, multiple recruitment strategies were employed to reach potential participants. The small sample size of n=75 respondents out of 1,399 professional dancers employed in German theaters [80] results in a response rate of 5.39% (RR1) and substantially limits the study's representativeness [81]. The findings should therefore be interpreted with caution and understood as specific to the sample rather than generalizable to the entire population of professional dancers in German theaters. Despite the explanation of the anonymity and the scientific framework, possible reasons for not participating may be the fear of being exposed due to asymmetric power structures. In addition, the questionnaire was pre-tested and optimized with several participants; however, its complexity may still have affected response quality. Although the WAI has not yet been applied to the professional dancer population, parallels can be drawn to its application in professions involving high levels of physical activity, such as nursing or healthcare workers with high work-related physical activity profiles [82–85]. With regard to the psychometric properties of the WAI, studies have found heterogeneous results [86, 87] and there is a potential bias due to self-assessment. Moreover, the WAI was originally designed for the general working population rather than for artists, and the development of a dance-specific assessment tool could provide greater precision in the future. But it should be noted that the WAI is very useful in practice due to its manageability and many proven applications [88]. While the empirical findings are embedded within German public theaters, the theoretical model identifies universal organizational dynamics including hierarchical management structures, work organization challenges, physical and psychological work demands, and performance competencies that characterize theater organizations internationally. This suggests that the model has broad applicability across cultural contexts, which warrants empirical validation in future research.

## Conclusions

The present study explores the barriers and opportunities for the implementation of measures to enhance the performance ability of professional dancers in theaters. The resulting model of integrated performance ability in professional dance reconceptualizes performance ability in professional dance as a dynamic, integrated outcome shaped by both individual and structural factors. This concept extends the traditional conception of work ability to encompass a biopsychosocial and context-dependent framework, emphasizing its multifaceted nature. It presents a comprehensive model for understanding and supporting integrated performance ability. This systemic perspective provides a valuable foundation for future research and practical applications in occupational health and dance-specific performance development. Furthermore, contextual factors, including the size of the ensemble and existing hierarchical structures, must be taken into account. It is imperative to allocate particular attention to specific vulnerable groups, such as Demi-soloists, in the formulation of empowerment strategies.

## Data Availability

To safeguard the anonymity of the participants, no interview transcripts or records can be made available. The second phase dataset may be requested from the corresponding author if the purpose is sufficiently justified.

## Abbreviations

WAI: Work Ability Index
WHO: World Health Organization
